# Fluoroquinolones and risk of nightmares: A literature review and disproportionality analysis using individual case safety reports from Food and Drug Administration Adverse Event Reporting System database

**DOI:** 10.1177/02698811251344684

**Published:** 2025-06-27

**Authors:** Mohammad Ali Omrani, Christian T Tsobo, Niaz Chalabianloo, Fatemeh Ahmadi, Sheikh S Abdullah, Flory T Muanda

**Affiliations:** 1 Department of Physiology and Pharmacology, Western University, London, ON, Canada; 2 Unit of Clinical Pharmacology and Pharmacovigilance, University of Kinshasa, Kinshasa, Democratic Republic of the Congo; 3 ICES Western, London, ON, Canada; 4 Department of Epidemiology and Biostatistics, Western University, London, ON, Canada; 5 Lawson Health Research Institute, London Health Sciences Center, London, ON, Canada

**Keywords:** Fluoroquinolones, nightmares, disproportionality analysis, pharmacovigilance

## Abstract

**Background:**

Fluoroquinolones (FQs) have been linked to various neuropsychiatric effects, including nightmares, mostly through case reports. However, data on nightmares remain limited and underreported.

**Aims:**

To review the literature on FQ-related nightmares and estimate the risk of nightmares associated with FQs compared to other antibiotics using data from the Food and Drug Administration Adverse Event Reporting System (FAERS) database.

**Methods:**

A literature review was conducted to identify studies on FQ-related nightmares. Active-comparator restricted disproportionality analyses were performed in FAERS (2004Q1–2023Q4) for ciprofloxacin, levofloxacin, and moxifloxacin compared to azithromycin and trimethoprim-sulfamethoxazole. We calculated reporting odds ratios (RORs), proportional reporting ratios, adjusted ROR (accounting age, sex, weight, and specific indications), and information components (IC_025_) to detect safety signals for the Medical Dictionary for Regulatory Activities term “nightmare.”

**Results:**

The review identified seven studies, with the prevalence of nightmares ranging from 0.01% to 8% across three trials. Disproportionality analyses indicated that FQ-associated nightmare reports were 6- to 10-fold higher than those linked to azithromycin (ROR: 6.18, 95% CI: 4.14–9.23) and trimethoprim-sulfamethoxazole (ROR: 10.38, 95% CI: 4.92–21.89), largely reported by consumers. These findings were consistent across frequentist and Bayesian methods and adjusted analyses.

**Conclusion:**

FQs may increase the risk of nightmares. Our findings provide valuable insights for future research on their safety profile. Further research is needed to validate these findings and guide safe FQ use.

## Introduction

Fluoroquinolones (FQs) are synthetic broad-spectrum antibiotics commonly used to treat gastrointestinal, respiratory, genitourinary, and ophthalmic infections (Redgrave et al., 2014). However, these medications have been linked to serious complications and disabilities despite their therapeutic benefits. FQ use has been associated with central nervous system (CNS) effects, including agitation, tremors, hallucinations, psychosis, and convulsions (Baggio and Ananda-Rajah, 2021; HealthCanada, 2017; Mandell and Tillotson, 2002). FDA’s 2018 warnings and precautions on FQ labels addressed psychiatric effects. In 2020, the Australian Therapeutic Goods Administration recommended that FQ labels be updated to include warnings about psychiatric adverse reactions, such as nightmares (FDA, 2013, 2016a, 2016b). However, the current evidence suggesting a potential link between FQ treatment and nightmares remains limited (Dey, 1995; Upton, 1994). To date, no real-world study has explored this association using individual case safety reports (ICSRs) from the US Food and Drug Administration Adverse Event Reporting System (FAERS). Our primary objective was to determine whether FQs are associated with a higher risk of nightmares compared to other antibiotics used for similar indications. To address this question, we conducted a literature review and performed an active-comparator restricted disproportionality analysis (ACR-DA) on FAERS to quantify the risk of nightmares linked to FQ use.

## Methods

### Literature review

We searched MEDLINE and EMBASE to identify relevant scientific literature from inception to March 4, 2025, with no language restrictions. The comprehensive search strategy, which included keywords related to FQs and nightmares, is detailed in the Supplemental material Tables S1 and S2. Our review included all published reports of FQ associated with nightmares, including case reports, case series, observational studies, and randomized clinical trials. To maintain a focus on empirical evidence, reviews, studies with no full-text availability, and in vitro studies were excluded.

The titles and abstracts of the articles identified through the literature search were reviewed, excluding those that did not meet the predetermined criteria. We then reviewed the full texts of the remaining articles to confirm their eligibility for inclusion in our analysis. Data extraction forms were standardized for each study included to ensure consistency. In the extracted data, authors, the year of publication, the study design, the location, population characteristics, the type of FQ used, the dosage, the time to nightmare onset, and nightmare-related outcomes were analyzed.

We applied the Naranjo scale to case reports specifically to examine the likelihood of nightmares being associated with FQ use. Observational studies were assessed using the Downs and Black checklist, while randomized clinical trials were assessed using the Risk of Bias 2 (ROB2) tool (Sterne et al., 2019).

### Disproportionality analysis using ICSRs from FAERS

An ACR-DA was conducted using ICSRs from the FAERS database (Alkabbani and Gamble, 2023). The ACR-DA method employed in this pharmacovigilance study is a specialized form of disproportionality analysis (DA), a widely used tool for detecting safety signals in pharmacovigilance. In standard DA, the frequency of a safety outcome reported for one medication is compared to the same outcome reported for all other medications in the database. In contrast, ACR-DA focuses specifically on comparing the safety outcomes of the medication under investigation (in this case, FQs) with those of an active comparator—another specific medication used in a similar clinical setting. False-positive signals can be reduced with this focused approach.

Our primary objective was to identify signals of disproportionality between nightmares associated with FQs, including ciprofloxacin, levofloxacin, and moxifloxacin, and two other antibiotics—azithromycin and trimethoprim-sulfamethoxazole. To achieve this, we compared the number of reported nightmares in FQ ICSRs to those in ICSRs for azithromycin and trimethoprim-sulfamethoxazole. Additionally, we evaluated whether there is any difference in nightmare reports across FQ ICSRs.

## FAERS database

### Data source

We utilized data from the (FAERS., 2024) accessed through the (OpenFDA., 2024) platform, which is publicly available (openFDA; FAERS). The study period encompassed reports submitted from the first quarter of 2004 to the fourth quarter of 2023. We selected OpenFDA for its partially preprocessed format, which reduces duplicates and provides a more structured dataset compared to raw FAERS data (Kass-Hout et al., 2015).

### Data extraction, cleaning, and processing

Data extraction was performed using a custom script to download JSON files for the study period. Despite the structured format provided by OpenFDA, extensive data cleaning was necessary due to inconsistencies in drug names, particularly in the “medicinal product” field. Multiple variations in drug names were identified during a manual review, increasing usable information. We used regular expressions to normalize these names during preprocessing and matched them against databases such as RxNorm and Observational Health Data Sciences and Informatics (Hripcsak et al., 2015). To further enrich the dataset, we added RxNorm Concept Unique Identifiers, generic names, and Anatomical Therapeutic Chemical classification system codes (Health., 2024). The drug characterization fields in OpenFDA were mapped to the role code field in FAERS, facilitating the identification of primary suspect drugs. Duplicate cases were identified and removed by cross-referencing safety report ID numbers linked to each report, ensuring the final dataset was clean and focused on primary suspects of adverse reactions.

### Exposed and comparator groups

The exposed group consisted of ICSRs, for which FQs, including ciprofloxacin, levofloxacin, and moxifloxacin, were identified as the primary suspects responsible for the adverse event. The comparator group included ICSRs for which azithromycin and trimethoprim-sulfamethoxazole were listed as the primary suspects for the adverse event. We selected azithromycin and trimethoprim-sulfamethoxazole as active comparators due to their similarity in indications to FQs (Supplemental material Table S3). Additionally, these antibiotics exhibit some degree of CNS penetration, although to a lesser extent than FQs (Althubyani et al., 2024; Haddad et al., 2022). To further account for potential differences in blood-brain barrier permeability, we also conducted within-class comparisons among FQs, which share similar pharmacokinetic properties and higher CNS penetration. This internal comparison assessed whether the observed disproportionality in nightmare reports is specific to FQs or attributable to broader antibiotic effects.

### Study outcome

We used the following Medical Dictionary for Regulatory Activities preferred terms—nightmares—to capture the study outcome.

### Statistical analysis

Disproportionality analyses were conducted using both frequentist measures, including the proportional reporting ratio (PRR) and reporting odds ratio (ROR), and Bayesian measures, such as the information component (IC). The PRR assesses the frequency of adverse events associated with the drug of interest compared to other medications in the database (Evans et al., 2001), while the ROR evaluates the odds of an adverse event occurring with the medication of interest versus other medications. The IC detects disproportionate reporting by calculating the deviation from expected values for specific drug-event combinations. We defined a signal by three criteria: an IC_025_ (the lower 2.5% confidence limit of the IC) greater than 0, a lower bound of the 95% confidence interval (CI) of the ROR exceeding 1.0, and at least three reported cases (Rothman et al., 2004; van Puijenbroek et al., 2002). Additionally, adjusted RORs were calculated using logistic regression, accounting for confounding factors such as sex, age category, weight category, and indications of respiratory and urinary tract infections.

Forest plots were generated to visualize the comparisons between FQs and the active comparators. All statistical analyses were performed using SAS (Version 8.3, SAS Studio, Cary, NC, USA) and R (Version 4.3.3, R Foundation for Statistical Computing, Vienna, Austria).

## Results

### Literature review

After removing duplicates, a total of 2600 studies were retrieved through database searches. Following the screening of titles and abstracts, we reviewed the full texts of 12 studies. From these, seven articles were included, comprising three papers highlighting four case reports, one cross-sectional study, and three clinical trials that reported nightmares as adverse drug events associated with FQs (a summary of the studies and flowchart of review are available in the Supplemental material Tables S4–S6 and Figure S1). In three included articles highlighting case reports, we identified four cases linking nightmares to FQ use (Supplemental material Table S4), with three of these cases (75%) involving young children. The median age of the four patients was 5.5 years (IQR: 4.75–15), and two (50%) were male. Nightmare symptoms in children were identified with the assistance of parents or caregivers. Among the cases in children, only one report specified the oral dose of ofloxacin (400 mg twice daily) for treating Pseudomonas aeruginosa infection. Another case involved a 24-year-old female who experienced nightmares after taking 1000 mg of ciprofloxacin per day. After experiencing nightmares, all four patients (100%) discontinued FQ treatment, with a median time to relief after discontinuation of 24 h (IQR: 12–24). Ciprofloxacin (75%) was the most frequently prescribed FQ, and Salmonella typhi (50%) was the most reported indication. Using the Naranjo algorithm, we established a probable causal relationship in one case report (score 9), while the other three reports indicated a possible causal relationship (score 7). The median time to adverse events was 2 days (IQR: 1–3) from FQ initiation (Dang et al., 2008; Dey, 1995; Upton, 1994).

In the cross-sectional study, which utilized a web-based survey to investigate neuropsychiatric events associated with FQ treatment, 21% of the 94 individuals receiving FQ treatment reported experiencing nightmares as a potential adverse drug event (Supplemental material Table S5; Kaur et al., 2016).

Among the three clinical trials, two were conducted in North America (Canada and the United States), and one was conducted in Europe (Germany). The prevalence of nightmares across these trials ranged from 0.01% to 8% (Table S6). In the first clinical trial, a double-blind study conducted in Canada, 7 out of 85 patients (8%) receiving varying doses of Fleroxacin, a long-acting FQ, reported experiencing nightmares (Bowie et al., 1989). The second clinical trial, comprising phase II–IV studies conducted in Germany, involved 10,298 patients treated with Ofloxacin, where only one case of nightmares (0.01%) was recorded (Blomer et al., 1986). In the third clinical trial, a phase III randomized trial comparing the efficacy and safety of solithromycin (*n* = 64 patients) with levofloxacin (*n* = 68 patients) for treating community-acquired bacterial pneumonia, one case of nightmares (1.5%) was reported among the 68 patients receiving Levofloxacin (Oldach et al., 2013).

### Clinical characteristics of cases of nightmares in FAERS

After removing duplicates, we identified 114,130 reports in the FAERS, with the primary suspect drugs being FQs, trimethoprim-sulfamethoxazole, and azithromycin. The total number of reports was 82,632 for FQs, 21,431 for azithromycin, and 10,067 for trimethoprim-sulfamethoxazole. Among the total reports of FQs, 593 cases of nightmares were documented, accounting for 0.7% of all FQ reports. The breakdown for specific FQs was 271 cases for ciprofloxacin, 255 for levofloxacin, and 67 for moxifloxacin. In comparison, the trimethoprim-sulfamethoxazole and azithromycin groups reported only 7 and 25 cases of nightmares, respectively.

The baseline clinical characteristics of nightmare reports related to FQs, azithromycin, and trimethoprim-sulfamethoxazole are presented in Table 1. The median age of individuals reporting nightmares was 46 years (IQR: 33–58) for ciprofloxacin, 47 years (IQR: 37–60) for levofloxacin, and 50 years (IQR: 35–63) for moxifloxacin. A significant portion of FQ users reporting nightmares were aged 18–65 years. In contrast, the median ages for azithromycin and trimethoprim-sulfamethoxazole users reporting nightmares were 37 (IQR: 14–72) and 19 [IQR: 13–58), respectively.

**Table 1.** Clinical characteristics of reports associated with fluoroquinolones, azithromycin, and trimethoprim-sulfamethoxazole-related nightmares from the FAERS database from 2004Q1 to 2023Q4.

Characteristics	Ciprofloxacin (*N*, %)	Levofloxacin (*N*, %)	Moxifloxacin (*N*, %)	All fluoroquinolones (*N*, %)	Azithromycin (*N*, %)	Trimethoprim-sulfamethoxazole (*N*, %)
Number of events of nightmares	271 (0.8)	255 (0.7)	67 (0.4)	593 (0.7)	25 (0.1)	7 (<0.1)
Age (year)						
Median age (IQR)	46 (33–58)	47 (37–60)	50 (35–63)	47 (35–60)	37 (14–72)	19 (13–58)
<18	4 (1.9)	3 (1.1)	0 (0)	7 (1.18)	4 (16.0)	2 (28.5)
18–< 45	94 (45.1)	88 (34.5)	20 (29.8)	202 (34.3)	3 (12.0)	1 (14.2)
45–<65	83 (30.9)	83 (32.5)	23 (34.3)	189 (32.1)	0 (0)	1 (14.2)
65–<100	30 (11.0)	35 (13.7)	8 (11.9)	73(12.3)	6 (24.0)	1 (14.2)
Unknown	60 (22.1)	46 (18.0)	16 (23.8)	122 (20.57)	12 (48.0)	2 (28.8)
Sex						
Male	92 (33.9)	53 (20.7)	12 (17.9)	157 (26.4)	11 (44.0)	0 (0)
Female	160 (59.0)	185 (72.5)	54 (80.6)	399 (67.2)	12 (48.0)	7 (100)
Unknown	19 (7.0)	17 (6.6)	1 (1.4)	37 (6.2)	2 (8.0)	0 (0)
Weight (kg)						
Median weight (IQR)	63.5 (57.6–72.9)	68.0 (58.9–81.6)	74.8 (54.4–90.7)	67.1 (57.6–77.1)	58.0 (51.2–63.0)	43.0 (13.1–81.6)
<43	0 (0)	3 (1.1)	1 (1.4)	4 (0.6)	2 (8.0)	1 (14.2)
43–<76	141 (52.0)	131 (51.3)	23 (34.3)	295 (49.9)	6 (24.0)	1 (14.2)
76–>92	11 (4.0)	36 (14.1)	11 (16.4)	58 (9.8)	0 (0)	1 (14.2)
92–>180	17 (6.2)	28 (10.9)	7 (10.4)	52 (8.7)	1 (4.0)	0 (0)
Unknown	102 (37.6)	57 (22.3)	25 (37.3)	184 (31.0)	16 (64.0)	4 (57.1)
Time to nightmare report (days)						
Median time to nightmare report (IQR)	5 (1–7)	4 (2–7)	3 (2–5)	5 (2–7)	4 (0–4)	33 (6–60)
Total number of available reports	158	169	37	364	13	2
<2	24 (15.1)	28 (16.5)	8 (21.6)	60 (16.4)	4 (30.7)	0 (0)
2–10	104 (65.8)	117 (69.2)	29 (78.3)	250 (68.6)	8 (61.53)	1 (50.0)
10–30	21 (13.3)	17 (10.0)	0 (0)	38 (10.4)	0 (0)	0 (0)
>30	9 (5.7)	7 (4.1)	0 (0)	16 (4.4)	0 (0)	1 (50.0)
Indication of use						
Urinary tract infection	114 (42.0)	36 (14.1)	3 (4.4)	153 (25.8)	0 (0)	1 (14.2)
Respiratory tract infection	19 (7.0)	113 (44.3)	42 (62.6)	174 (29.34)	11 (44.0)	0 (0)
Unknown	23 (8.4)	12 (4.7)	7 (10.4)	42 (7.0)	3 (12.0)	1 (4.0)
Reported person						
Physician	21 (8.7)	17 (8.7)	4 (7.2)	42 (8.5)	2 (10.5)	1 (20.0)
Pharmacist	5 (2.0)	13 (6.7)	2 (3.6)	20 (4.0)	6 (31.5)	0 (0)
Other health professional	55 (22.8)	19 (9.7)	18 (32.7)	92 (18.7)	3 (15.7)	2 (40.0)
Lawyer	1 (0.4)	4 (2.0)	0 (0)	5 (1.0)	0 (0)	0 (0)
Consumer of non-health professional	159 (65.9)	141 (72.6)	31 (56.3)	331 (67.5)	8 (42.1)	2 (40.0)
Unknown	30 (11.0)	61 (23.9)	12 (17.9)	103 (17.3)	6 (24.0)	0 (0)
Reporting country						
United States	104 (38.3)	196 (76.8)	46 (68.6)	346 (58.3)	9 (36.0)	4 (57.1)
Canada	13 (4.8)	13 (5.1)	7 (10.4)	33 (5.5)	3 (12.0)	0 (0)
Great Britain	99 (36.5)	7 (2.7)	2 (2.3)	108 (18.2)	0 (0)	0 (0)
Germany	29 (10.7)	12 (4.7)	1 (1.5)	42 (7.1)	0 (0)	0 (0)
Rest of world	21 (7.7)	16 (6.2)	6 (8.9)	43 (7.2)	8 (32.0)	1 (14.2)
Unknown	5 (1.8)	11 (4.3)	5 (7.4)	21 (3.5)	5 (20.0)	2 (28.57)
Serious outcome						
Death	4 (1.4)	0 (0)	0 (0)	4 (0.6)	0 (0)	0 (0)
Disabling	30 (11.0)	31 (12.1)	4 (5.9)	65 (10.9)	0 (0)	0 (0)
Hospitalization	14 (5.1)	7 (2.7)	5 (7.4)	26 (4.3)	2 (8.0)	0 (0)
Life-threatening	7 (2.5)	20 (7.8)	6 (8.9)	33 (5.5)	0 (0)	1 (14.2)
Not serious	29 (10.7)	54 (21.1)	13 (19.4)	96 (16.1)	8 (32.0)	2 (28.5)
Other	187 (69.0)	143 (56.0)	39 (58.2)	369 (62.2)	15 (60.0)	4 (57.1)

IQR: interquartile; FAERS: US Food and Drug Administration Adverse Event Reporting System.

Sex distribution indicated that females accounted for a majority of nightmare reports related to FQ use, with 59% of ciprofloxacin cases, 72.5% of levofloxacin cases, and 80.6% of moxifloxacin cases being female. In contrast, the sex distribution among azithromycin users was nearly balanced, while all patients who reported nightmares associated with trimethoprim-sulfamethoxazole were women.

Data on the time to nightmare report were available for 364 consumers for each FQ, as shown in Table 1. Among FQs, moxifloxacin had the shortest median time to report nightmares at 3 days (IQR: 2–5), followed by levofloxacin at 4 days (IQR: 2–7) and ciprofloxacin at 5 days (IQR: 1–7). Most nightmares were reported within a timeframe of 2–10 days after starting the medications. For azithromycin, the median report time was 4 days (IQR: 0–4), while trimethoprim-sulfamethoxazole had a substantially longer median report time of 3 days (IQR: 6–60).

Respiratory tract infections were more prevalent among levofloxacin (44.3%) and moxifloxacin (62.6%) users, while urinary tract infections were the most common indication for ciprofloxacin (42.0%). Conversely, a much lower percentage of consumers using azithromycin and trimethoprim-sulfamethoxazole reported these indications.

Consumers reported more cases than healthcare professionals. Consumers submitted 67.5% of nightmare reports for FQs, while healthcare professionals, including physicians and pharmacists, submitted 31.2%. For azithromycin and trimethoprim-sulfamethoxazole, 42.1% and 40%, respectively, were submitted by consumers.

Nightmare reports predominantly originated from the United States, accounting for 58.3%, 36.0%, and 57.1% of FQs, azithromycin, and trimethoprim-sulfamethoxazole reports. Serious outcomes, including death, hospitalization, life-threatening events, and disability, were reported, with disability being the most frequent serious outcome at 10.9%. However, most cases were not classified as serious.

### Active-comparator restricted disproportionality analyses in FAERS

When azithromycin and trimethoprim-sulfamethoxazole served as active comparator groups for FQs as a class and individual FQs, the PRR, ROR, adjusted ROR, and IC for FQs indicated significantly higher reports of nightmares compared to azithromycin and trimethoprim-sulfamethoxazole in the ACR-DA within FAERS (Table 2). FQs showed approximately 6-fold and 10-fold higher nightmare reports than azithromycin and trimethoprim-sulfamethoxazole. The IC_025_ values further supported these findings, with values of 0.15 and 0.03 when azithromycin and trimethoprim-sulfamethoxazole were used as active comparator groups.

**Table 2.** Reporting odds ratio (ROR), adjusted reporting odds ratio (aROR), proportional reporting ratio (PRR) with 95% CI, information component (IC), and IC_025_ of nightmare reports for fluoroquinolones in comparator analyses in FAERS.

Drug of interest	Comparators	ROR (95% CI)	aROR (95% CI)^a^	PRR (95% CI)	IC (IC_025_)
Ciprofloxacin	Azithromycin	6.94 (4.61–10.46)	9.76 (4.52–21.04)	6.90 (4.58–10.39)	0.58 (0.41)
	Trimethoprim-sulfamethoxazole	11.66 (5.50–24.70)	12.33 (3.92–38.75)	11.57 (4.46–24.52)	0.34 (0.16)
	Other fluoroquinolones	1.22 (1.04–1.44)	0.91 (0.71–1.17)	1.22 (1.04–1.43)	0.17 (−0.01)
Levofloxacin	Azithromycin	6.69 (4.37–10.09)	11.86 (5.69–24.71)	6.64 (4.40–10.03)	0.59 (0.41)
	Trimethoprim-sulfamethoxazole	11.23 (5.30–23.81)	16.38 (5.18–51.77)	11.15 (5.26–23.64)	0.35 (0.16)
	Other fluoroquinolones	1.14 (0.97–1.34)	1.40 (1.12–1.75)	1.14 (0.97–1.34)	0.14 (−0.04)
Moxifloxacin	Azithromycin	3.57 (2.25–5.66)	4.55 (2.02–10.26)	3.56 (2.25–5.65)	0.76 (0.39)
	Trimethoprim-sulfamethoxazole	6.00 (2.75–13.08)	7.74 (1.99–30.02)	5.98 (2.74–13.04)	0.55 (0.19)
	Other fluoroquinolones	0.52 (0.40–0.67)	0.55 (0.38–0.79)	0.52 (0.40–0.67)	−0.78 (−1.2)
All fluoroquinolones^b^	Azithromycin	6.18 (4.14–9.23)	9.67 (4.74–19.72)	6.15 (4.12–9.18)	0.27 (0.15)
	Trimethoprim-sulfamethoxazole	10.38 (4.92–21.89)	12.96 (4.15–40.42)	10.32 (4.89–21.74)	0.15 (0.03)

ROR: reported odds ratio; CI: confidence interval; aROR: adjusted reported odds ratio; PRR: proportional reporting ratio; IC: information component.

a Adjusted by sex, age category, weight category, urinary tract infection indication, and respiratory tract infection indication.

b Nightmare reports associated with ciprofloxacin, levofloxacin, and moxifloxacin.

A safety signal for nightmares was identified with ciprofloxacin compared to azithromycin, showing an ROR of 6.94 (95% CI: 4.61–10.46) and an IC_025_ of 0.41. Similarly, ciprofloxacin reported 11-fold higher nightmares reports than trimethoprim-sulfamethoxazole (ROR: 11.66, 95% CI: 5.50–24.70; IC_025_ 0.16). Comparable disproportionality in nightmare reports was also observed for levofloxacin and moxifloxacin compared to azithromycin and trimethoprim-sulfamethoxazole (Table 2).

**Figure 1.**
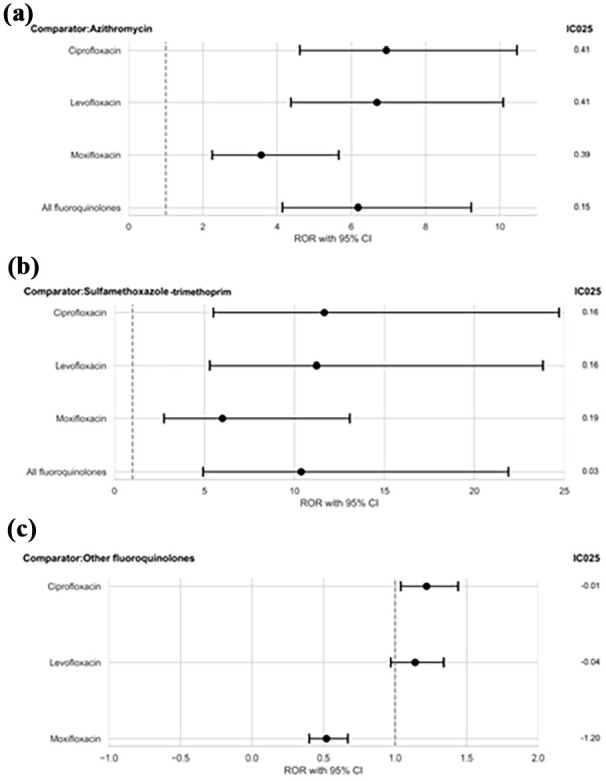
ROR forest plots with IC_025_ for active-comparator restricted disproportionality analyses of nightmares for ciprofloxacin, levofloxacin, and moxifloxacin compared to (a) azithromycin, (b) trimethoprim-sulfamethoxazole, and (c) other fluoroquinolones in FAERS.

When non-ciprofloxacin FQs served as an active comparator group, ciprofloxacin showed a higher ROR of 1.22 (95% CI: 1.04–1.44) compared to other FQs, with an IC_025_ of −0.01. Consistent results were obtained with the adjusted ROR and PRR across all analyses. ROR forest plots with IC_025_ for each analysis with the active comparator group are also presented in Figure 1.

## Discussion

We systematically reviewed the available literature and conducted a DA using the FAERS database to examine the risk of nightmares associated with FQs. Seven studies were included in the review. The prevalence of nightmares ranged from 0.01% to 8% across three trials. Among the three included trials, only one compared levofloxacin with solithromycin. However, nightmares were not reported in patients receiving solithromycin. Furthermore, the cross-sectional internet-based survey, in which 21% of FQ users reported nightmares, was conducted within the Floxed Network, a social network of FQ-treated persons who reported long-term neuropsychiatric toxicity. Due to its uncontrolled design and recruitment from a network predisposed to negative perceptions of FQs, the study is susceptible to selection and reporting biases. Moreover, several limitations, including small sample sizes, short follow-up durations, and incomplete data for assessing contributing factors, should be taken into account when interpreting these results. Additionally, the studies were not specifically designed to examine the risk of nightmares, which may have led to underreporting of this adverse event.

Our DA showed that nightmare reports associated with FQs were 6- to 10-fold higher than those linked to other antibiotics, such as azithromycin and trimethoprim-sulfamethoxazole. These results indicate a potential safety signal for nightmares related to FQs, particularly ciprofloxacin. Notably, most cases of nightmares were reported by consumers. Given that nightmares might not always be perceived as serious by consumers, this underreporting by healthcare professionals highlights the importance of patient-reported outcomes in capturing adverse events that may otherwise go unnoticed in clinical settings.

These findings align with previous reports suggesting that neuropsychiatric side effects, including nightmares, may be associated with FQs, particularly ciprofloxacin. While some earlier studies have noted neuropsychiatric effects such as anxiety, hallucinations, and confusion, few have specifically focused on nightmares, and our analysis provides a more detailed understanding of this association. The observed prevalence of nightmares in our review is consistent with existing literature, which highlights a wide variability in neuropsychiatric adverse effects (Hublin et al., 1999; Janson et al., 1995). FQs are commonly prescribed for a wide range of bacterial infections, and their known adverse effects primarily involve tendinopathies and gastrointestinal disturbances. However, the potential for neuropsychiatric adverse drug events has garnered increasing attention, with reports highlighting the need for awareness of such reactions among prescribers and patients. Our review contributes to this growing body of evidence by providing a focused evaluation of nightmares as an underrecognized but significant adverse effect of FQ therapy.

The mechanisms underlying these neuropsychiatric effects, including nightmares, are not yet fully understood, though they may involve FQs’ ability to cross the blood-brain barrier and affect CNS pathways (Moorthy et al., 2008; Wierzbiński et al., 2023). Further research is needed to elucidate these mechanisms and to identify patients who may be particularly vulnerable to such adverse effects, such as those with pre-existing psychiatric conditions or compromised renal function. One proposed mechanism involves FQs interacting with gamma-amino butyric acid (GABA) receptors in the CNS. Since GABA is the main inhibitory neurotransmitter, its blockage can lead to increased neuronal excitability and altered sleep patterns, including nightmares (Kawakami et al., 1997; Pagel and Helfter, 2003). Furthermore, research has suggested that antibiotics, including FQs, might trigger immune responses that could contribute to the onset of nightmares (Pagel and Helfter, 2003). Given the common occurrence of nightmares in the general population, establishing a direct causal link between FQ use and nightmares can be challenging (Dang et al., 2008). However, recognizing nightmares as a potential adverse drug event is crucial for clinical practice, especially for patients with pre-existing psychiatric conditions or those reporting unusual sleep disturbances.

Several limitations are associated with spontaneous reporting systems, including underreporting and reporting biases. Notably, a significant proportion of the nightmare reports in our study were submitted by consumers rather than healthcare professionals. Additionally, while disproportionality analyses can detect safety signals and suggest research hypotheses, the observational nature of the data limits our ability to determine causality (Mouffak et al., 2021). The potential influence of underlying infections on neuropsychiatric effects in FQ users may also confound the relationship between FQ use and nightmares (Munjal et al., 2017). For example, FQs are often used to treat bacterial infections that themselves may contribute to fever and neuropsychiatric symptoms, such as nightmares, which can distort the observed association. However, by comparing the frequency of nightmares in FQ users with other medications that share similar indications, we reduce the concern about confounding by indication, as the comparison allows for better differentiation of the drug-specific effects from those driven by the underlying infections. There are also additional biases inherent in spontaneous reporting data that could affect the findings. Notoriety bias could occur if increased media attention or regulatory warnings about FQs lead to more frequent reporting of nightmares, even if the actual risk remains unchanged. Selection bias may arise from the focus on primary suspect cases, as only those who reported nightmares as the main adverse event were included. This approach enhances specificity and reduces noise from reports that involve secondary suspects or concomitant medications. However, this exclusion of cases with secondary suspect drugs or those with interacting medications could potentially mask relevant associations or may have contributed to the observed signal. Despite this limitation, we feel more confident in the robustness of the observed signal, given the clear association with primary suspect drugs and the consistency of our results across analytical methods. Additionally, data quality biases related to missing or incomplete demographic and clinical data, such as the baseline cognitive function of patients, may distort the analysis and introduce inaccuracies in interpreting the results. However, we adjusted for reported FQ indications, which may have reduced the risk of bias related to underlying conditions or inappropriate comparisons.

Finally, we acknowledged the potential impact of FQs’ ability to cross the blood-brain barrier and cause neuropsychiatric symptoms such as nightmares. While other antibiotics, such as trimethoprim-sulfamethoxazole and azithromycin, do not share the same pharmacokinetic profile or cross the blood-brain barrier as effectively, we have taken this into account in our study design and within-class comparisons.

In conclusion, our study showed that FQs may be associated with an increased risk of nightmares. While acknowledging the limitations of spontaneous reporting databases and the studies included in our review, our findings offer important insights and serve as a foundation for future investigations into the safety profile of FQs. Further pharmacoepidemiologic research is needed to validate these findings.

## Supplemental Material

sj-docx-1-jop-10.1177_02698811251344684 – Supplemental material for Fluoroquinolones and risk of nightmares: A literature review and disproportionality analysis using individual case safety reports from Food and Drug Administration Adverse Event Reporting System database Supplemental material, sj-docx-1-jop-10.1177_02698811251344684 for Fluoroquinolones and risk of nightmares: A literature review and disproportionality analysis using individual case safety reports from Food and Drug Administration Adverse Event Reporting System database by Mohammad Ali Omrani, Christian T Tsobo, Niaz Chalabianloo, Fatemeh Ahmadi, Sheikh S Abdullah and Flory T Muanda in Journal of Psychopharmacology
