# Supplementary material for: Fluoroquinolones and risk of nightmares: A literature review and disproportionality analysis using individual case safety reports from Food and Drug Administration Adverse Event Reporting System database
Source: J Psychopharmacol. 2025 Jun 27;39(8):782–9. doi: 10.1177/02698811251344684 (PMC12287549; doi:10.1177/02698811251344684)
Supplement: sj-docx-1-jop-10.1177_02698811251344684 – Supplemental material for Fluoroquinolones and risk of nightmares: A literature review and disproportionality analysis using individual case safety reports from Food and Drug Administration Adverse Event Reporting System database [file sj-docx-1-jop-10.1177_02698811251344684.docx]

# The supplementary material for:

# Fluoroquinolones and Risk of Nightmares: A Literature Review and Disproportionality Analysis Using Individual Case Safety Reports from FAERS Database

Mohammad Ali Omrani^1^, Christian T Tsobo^2^, Niaz Chalabianloo^1^, Fatemeh Ahmadi^3,5^, Sheikh S. Abdullah^3,4^, Flory T Muanda ^2,3,4,5^

^1^ Department of Physiology and Pharmacology, Western University, London, Ontario, Canada,

^2^ Unit of Clinical Pharmacology and Pharmacovigilance, University of Kinshasa, Kinshasa, Democratic Republic of Congo,

^3^ ICES Western, London, Ontario, Canada,

^4^ Lawson Health Research Institute, London Health Sciences Centre, London, Ontario, Canada,

^5^Department of Epidemiology & Biostatistics, Western University, London, Ontario, Canada

**Corresponding Author**: Dr. Flory T Muanda, Department of Physiology and Pharmacology, Medical Sciences Building, 1151 Richmond St, Room 287, London, Ontario, Canada N6A 5C1 Tel: 519-661-2111-extension 84885,

Email: [fmuandat@uwo.ca](mailto:fmuandat@uwo.ca)

**Running title**: Fluoroquinolones and Risk of Nightmares

[**Table S1**](#tab2). A literature search in MEDLINE

**[Table S2](#tab3)** A literature search in Embase

**[Table S3](#tab1)** Comparison of labeled indications for ciprofloxacin, levofloxacin, moxifloxacin, azithromycin, and sulfamethoxazole according to UpToDate

[**Table S4.**](#tab4) Summary of case reports linking fluoroquinolones to nightmares

[**Table S5.**](#tab5)  Observational Studies reported fluoroquinolone-induced nightmares in patients.

[**Table S6.**](#tab6) Clinical trials reported fluoroquinolone-induced nightmares in patients.

[**Figure S1.**](#fig1) PRISMA flow chart of studies

[**References**](#_e_References)

| Table S1. A literature search in MEDLINE (1946 to March 4, 2025) | |
| --- | --- |
| 1 | fluoroquinolones/ or exp ciprofloxacin/ or exp gatifloxacin/ or exp gemifloxacin/ or exp moxifloxacin/ or exp norfloxacin/ or exp ofloxacin/ |
| 2 | (fluoroquinolon* or ciprofloxacin? or norfloxacin? or gatifloxacin? or gemifloxacin? or moxifloxacin? or norfloxacin? or ofloxacin? or levofloxacin?).tw. |
| 3 | 1 or 2 |
| 4 | (nightmare? or dream? or sleep).mp. |
| 5 | 3 and 4 |

| Table S2. A literature search in Embase (1947 to March 4, 2025) | |
| --- | --- |
| 1 | exp quinolone derivative/ or exp ciprofloxacin/ or exp gatifloxacin/ or exp moxifloxacin/ or exp norfloxacin/ |
| 2 | (fluoroquinolon* or ciprofloxacin? or norfloxacin? or gatifloxacin? or gemifloxacin? or moxifloxacin? or norfloxacin? or ofloxacin? or levofloxacin?).tw. |
| 3 | 1 or 2 |
| 4 | (nightmare? or dream? or sleep).mp. |
| 5 | 3and 4 |

| Table S3. Comparison of labeled indications for ciprofloxacin, levofloxacin, moxifloxacin, azithromycin, and sulfamethoxazole according to UpToDate | |
| --- | --- |
| **Drug** | **Labeled Indications** |
| Ciprofloxacin | Urinary tract infections, acute uncomplicated cystitis in females, chronic bacterial prostatitis, bone and joint infections, complicated intra-abdominal infections (with metronidazole), infectious diarrhea, typhoid fever, hospital-acquired pneumonia |
| Levofloxacin | Community-acquired pneumonia (CAP), nosocomial pneumonia, chronic obstructive pulmonary disease (COPD), acute bacterial rhinosinusitis, prostatitis, urinary tract infections, pyelonephritis, skin and skin structure infections, anthrax, plague |
| Moxifloxacin | Community-acquired pneumonia (CAP), acute bacterial rhinosinusitis, skin and skin structure infections, complicated intra-abdominal infections, plague |
| Azithromycin | Chancroid, COPD exacerbation, MAC (Mycobacterium avium complex) prevention, otitis media, pelvic inflammatory disease, community-acquired pneumonia (CAP), skin and skin structure infections, streptococcal pharyngitis |
| Sulfamethoxazole- Trimethoprim | Urinary tract infections, otitis media, chronic obstructive pulmonary disease exacerbations, Pneumocystis pneumonia (PCP), traveler’s diarrhea, shigellosis |

| Table S4. Summary of case reports linking fluoroquinolones to nightmares | | | | | | | | | | |
| --- | --- | --- | --- | --- | --- | --- | --- | --- | --- | --- |
| **Author /Year** | **Study Design** | **Age(year)** | **Sex** | **FQ-administered** | **Indication for FQ use** | **Dose (mg/day)** | **Days to nightmares ^a^** | **Treatment** | **Time to relief after DC** | **Naranjo score ^b^** |
| Dang et al., 2008(1) | Case Report | 24 | Female | Ciprofloxacin | Severe gastroenteritis | 1000 mg | 2 | DC of FQ | 1 day | 9 |
| Dey, 1995(2) | Case Report | 4.5 | Male | Ciprofloxacin | Fever, Salmonella typhi | Not specified | 1 | DC of FQ | 1 day | 7 |
|  | Case Report | 5 | Female | Ciprofloxacin | Fever, Salmonella typhi | Not specified | 3 | DC of FQ | 12 hours | 7 |
| Upton, 1994(3) | Case Report | 6 | Male | Ofloxacin (in addition to colistin) | Treating Pseudomonas aeruginosa | 800 mg | Not specified (returned after two weeks complaining of nightmares | DC of FQ | Not specified | 7 |

**Abbreviations**: FQ; Fluoroquinolone, DC; Discontinue

^a^ The median [IQR] days to nightmares was 2 (1 to 3)

^b^ Naranjo Adverse Drug Reaction Probability Interpretation: ≥ 9 = definite ADR, 5-8 = probable ADR, 1-4 = possible ADR, 0 = doubtful ADR.

| Table S5. Observational Studies reported fluoroquinolone-induced nightmares in patients. | | | | | | |
| --- | --- | --- | --- | --- | --- | --- |
| **Author /Year** | **Study Description** | **Study Type** | **Study Procedure/Exposure Time** | **Result on nightmares** | **Limitations** | **Quality Score ^a^** |
| Kaur et al., 2016(4) | 94 individuals from the Floxed Network, a social network of FQ-treated persons who reported long-term neuropsychiatric toxicity. | Web-based: Participants described their neuropsychiatric events and whether they reported these events to the FDA. | Web-based survey of FQ-treated persons | 21% of survey respondents reported nightmares | Self-reported survey data, no clinical validation, limited to web-based respondents | 9 |

^a^ We utilized the Modified Downs and Black checklist to evaluate the methodological quality of this study. We gave a score from 0 to 28, grouped into the following four quality levels: excellent (26 to 28), good (20–25), fair (15–19) and poor (14 or less).

| Table S6. Clinical trials reported fluoroquinolone-induced nightmares in patients. | | | | | | | | | | | |
| --- | --- | --- | --- | --- | --- | --- | --- | --- | --- | --- | --- |
| **Author /Year** | **Location** | **Sample Size** | **Population** | **Inclusion Criteria** | **Exclusion Criteria** | **Intervention** | **Control** | **Primary and**  **Secondary Outcome** | **Results on nightmares** | **Risk of Bias ^a^** | **Limitations** |
| Oldach et al. (2013)(5) | United States and Canada | 132 (65 randomized to solithromycin (64 received), 67 randomized to levofloxacin (68 received)) | 55.6 years (18-87 years), 49.2% Female | Adults ≥18 years with PORT risk class II, III, or IV, at least 3 of the following: cough with purulent sputum, dyspnea or tachypnea, chest pain, fever, rales, or evidence of pulmonary consolidation. No prior antibacterial therapy for current CABP unless treatment failure. Chest radiograph or CT showing new lobar or multilobar infiltrates consistent with acute bacterial pneumonia. | PORT risk class I, ventilator-associated pneumonia, anatomical bronchial obstruction, history of bronchiectasis, stage IV COPD, etc. | Solithromycin 800 mg on day 1, 400 mg daily on days 2-5 | Levofloxacin 750 mg daily on days 1-5 | Clinical success at test-of-cure visit  Early clinical response at day 3, microbiological success at test-of-cure | One patient in the levofloxacin group experienced nightmares, while no such events were reported in the solithromycin group | Low | Small sample size, short follow-up duration, low microbial isolation rate |
| Blomer et al., 1986(6) | Germany | 10,298 patients treated with ofloxacin. In phase III/IV | Male/Female ratio 1.3 | Patients were analyzed concerning age, sex, diagnosis of infection, underlying diseases, aggravating and complicating factors, ofloxacin dosage, duration of treatment, and type and severity of adverse reactions | Not mentioned | Ofloxacin | Not mentioned | Report adverse drug reactions | 1 report of nightmare between patients recorded in phase III/IV | High | Incomplete data to assess contributing factors, rare occurrence of symptoms |
| Bowie et al. , 1989(7) | Canada | 85 (28 received 400 mg, 29 received 600 mg, 28 received 800 mg) | 27.2 years (18-68 years), 27.1% Female | Individuals 18 years of age and older with uncomplicated genital infections, ambulatory setting. | Use of antacids, caffeine interaction, and sun exposure without precautions. | Fleroxacin 400 mg, 600 mg, or 800 mg once daily for 7 days | Not specified | Incidence of adverse reactions  Dose-related development of adverse reactions, correlation with outdoor occupation | 7 patients (9%) experienced bad dreams or hallucinations | Some concerns | Small sample size, lack of specific control group, high rate of adverse reactions at higher doses |

**Abbreviations:** PORT: Pneumonia Outcomes Research Team, COPD: Chronic Obstructive Pulmonary Disease, CABP: Community-Acquired Bacterial Pneumonia

^a^ The Risk of Bias 2 (ROB 2) tool was utilized for assessing the risk of bias with five domains: randomization process, deviations from intended interventions, missing outcome data, measurement of outcomes, and selection of reported results.


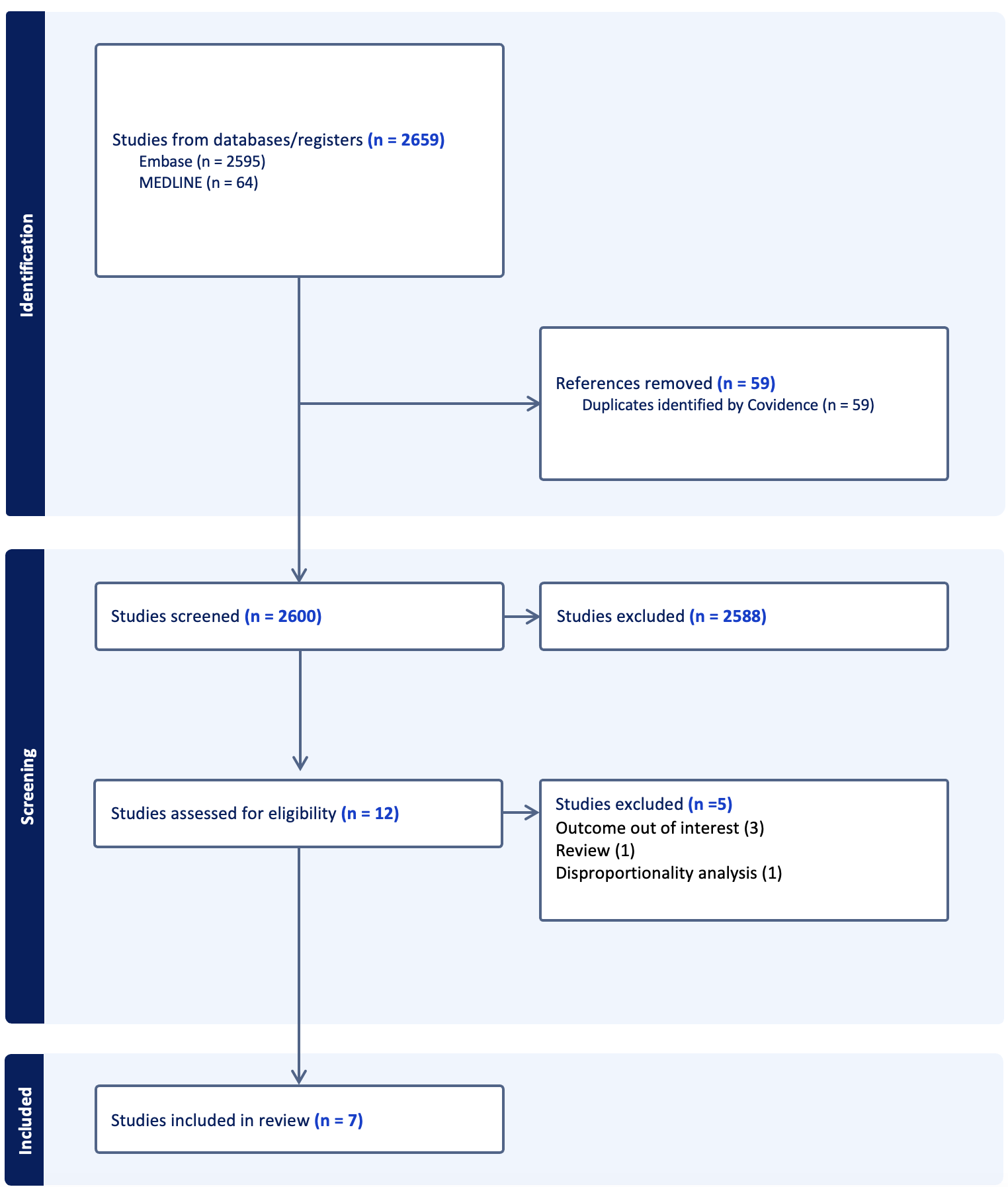


Figure S1. PRISMA flow chart of studies

1. Dang A, Kamat R, Padmanabh RV. Ciprofloxacin induced nightmares in an adult patient. Indian Journal of Psychiatry. 2008;50(4):305-6.

2. Dey S. Nightmare due to ciprofloxacin in young patients. Indian pediatrics. 1995;32:918-.

3. Upton C. Drug Points: Sleep disturbance in children treated with ofloxacin. BMJ. 1994;309(6966):1411.

4. Kaur K, Fayad R, Saxena A, Frizzell N, Chanda A, Das S, et al. Fluoroquinolone-related neuropsychiatric and mitochondrial toxicity: a collaborative investigation by scientists and members of a social network. The Journal of community and supportive oncology. 2016;14(2):54-65.

5. Oldach D, Clark K, Schranz J, Das A, Craft JC, Scott D, et al. Randomized, double-blind, multicenter phase 2 study comparing the efficacy and safety of oral solithromycin (CEM-101) to those of oral levofloxacin in the treatment of patients with community-acquired bacterial pneumonia. Antimicrobial agents and chemotherapy. 2013;57(6):2526-34.

6. Blomer R, Bruch K, Krauss H, Wacheck W. Safety of ofloxacin—adverse drug reactions reported during phase-II studies in Europe and in Japan. Infection. 1986;14(Suppl 4):S332-S4.

7. Bowie WR, Willetts V, Jewesson P. Adverse reactions in a dose-ranging study with a new long-acting fluoroquinolone, fleroxacin. Antimicrobial agents and chemotherapy. 1989;33(10):1778-82.

# References
